# Comparative Analysis of the Incidence of Selected Sexually Transmitted Viral Infections in Poland in 2010–2015: A Retrospective Cohort Study

**DOI:** 10.3390/jcm11123448

**Published:** 2022-06-15

**Authors:** Magda Orzechowska, Mateusz Cybulski, Elzbieta Krajewska-Kulak, Marek Sobolewski, Agnieszka Gniadek, Wiaczeslaw Niczyporuk

**Affiliations:** 1 Department of Epidemiology of Infectious Diseases and Supervision, National Institute of Public Health/National Research Institute, 00-791 Warsaw, Poland; 2 Department of Integrated Medical Care, Faculty of Health Sciences, Medical University of Bialystok, 15-096 Bialystok, Poland; mateusz.cybulski@umb.edu.pl (M.C.); elzbieta.krajewska-kulak@umb.edu.pl (E.K.-K.); 3 Department of Quantitative Methods, Rzeszow University of Technology, 35-959 Rzeszow, Poland; msobolew@prz.edu.pl; 4 Institute of Nursing and Midwifery, Faculty of Health Sciences, Jagiellonian University Medical College, 31-501 Krakow, Poland; agnieszka.gniadek@uj.edu.pl; 5 Department of Medical Sciences, Faculty of Health Sciences, Lomza State University of Applied Sciences, 18-400 Lomza, Poland; wniczyporuk@ansl.edu.pl

**Keywords:** genital herpes, genital warts, human immunodeficiency virus (HIV), sexually transmitted diseases, venereal diseases

## Abstract

Sexually transmitted infections (STIs) represent a major cause of morbidity in women and men worldwide. The main aim of this study was to perform a comparative analysis of the incidence of sexually transmitted viral infections in 2010–2015 in Poland, taking into account the administrative division of the country into provinces. This was a retrospective study. The analysed data came from the Centre for Health Information Systems of the Ministry of Health and the National Institute of Public Health-National Research Institute and constituted information from the epidemiological surveillance system in Poland. We collected data on the incidence of the following diseases: genital herpes (HSV), genital warts, human immunodeficiency virus (HIV) infection and acquired immunodeficiency syndrome (AIDS). The key groups with the highest risk of infection were young people between 20 and 29 years of age. The reported data on the incidence of genital herpes in Poland (*n* = 3378; 1.5/100,000) showed a downward trend, which does not coincide with global trends. Genital warts were the most frequent genital infections in Poland (*n* = 7980; 3.46/100,000), with significant regional variation. Over the analysed period, the situation of newly detected HIV infections seemed to be stable (*n* = 7144; 3.1/100,000). The incidence of these infections appeared to be highly correlated with urbanisation rates, which was not confirmed in the case of other analysed infections. The worsening epidemic situation with respect to sexually transmitted infections, the inefficiency of the current surveillance system and the reduction in funding for diagnosis and prevention, combined with inadequate legal solutions, make it necessary to undertake new legal and organisational measures aimed at improving the reproductive health in Poland in terms of sexually transmitted infections.

## 1. Introduction

Sexually transmitted infections (STIs) are a serious public health problem worldwide. This definition is used to refer to various clinical syndromes caused by pathogens that can be acquired and transmitted through sexual activity [1]. Almost 300 million people are infected with HPV, which can cause cervical cancer if persistent [2]. It is estimated that over 400 million people are infected with HSV-2, which causes genital herpes [3]. According to the United Nations Programme on HIV/AIDS (UNAIDS), more than 37 million people are living with HIV, which can cause acquired immunodeficiency syndrome (AIDS) [4].

Investigating and reporting the prevalence of sexually transmitted viral infections is important to implement or improve a wide range of health policies and programmes worldwide. To date, more than 30 microorganisms have been confirmed to be transmitted through sexual contact, including herpes simplex virus type 2 (HSV-2), human papillomavirus (HPV) and human immunodeficiency virus (HIV). The World Health Organization (WHO) estimates indicate that more than one million sexually transmitted infections occur every day [5].

While HPV vaccination was not obligatory in Poland, they are the so-called recommended vaccinations. They are financed by local self-government units under health policy programmes or from household budgets. In the years 2009–2016, as a part of local health policy, a total of 1204 health policy programs covering HPV vaccinations were implemented. As a part of these programs, 2.05% of girls, aged 10–14, were vaccinated [6].

The aim of this study was to perform a comparative analysis of the incidence of sexually transmitted viral infections (STVIs) between 2010 and 2015 in Poland.

## 2. Materials and Methods

### 2.1. Study Population and Design

This was a retrospective study. We performed a detailed comparative analysis of STVI incidence rates between 2010 and 2015, taking into account socio-demographic variables such as age and gender. We analysed data on STVIs (genital herpes, genital warts and HIV infections) registered under national epidemiological surveillance.

Our study was based on data from Polish public statistics on sexually transmitted infections. The analysed data came from the Centre of Health Information Systems of the Ministry of Health and provincial offices. The Yearbooks of the National Institute of Public Health-National Research Institute were an additional source of data on the incidence of HIV. These diseases are subject to annual reporting within the System of Public Statistics in Poland. The analysed data were sourced from collective annual reports on patients treated in dermatology and venerology outpatient clinics (MZ-14) from 16 provinces, sent by the service providers to provincial offices at a given site providing health services. The available data concerned the number of cases (by sex) in 9 age groups. The analysed data concerned the number of people treated in outpatient specialist medical centres in the field of venereology on the basis of a medical diagnosis. There were no reporting guidelines verifying the basis of diagnosis, such as symptoms or laboratory tests. This was described in the limitations of the study. The data obtained as part of routine surveillance was pseudonymised and transferred only as an annual aggregate number of people treated for STIs. Data are transferred by service providers to provincial offices, where they are then processed and, in the form of collective reports from the region, transferred to the Centre of Health Information Systems of the Ministry of Health. The data is processed digitally on the platform of the Ministry of Health for the national system of official statistics. In the case of data on HIV infection, they come from the National Institute of Public Health-National Research Institute.

The localisation of illness lesions was not included in the study because the collection system of information for sexually transmitted infections in Poland does not take into account such types of data. Moreover, the epidemiological reporting system for sexually transmitted infections in Poland does not include hepatitis B and hepatitis C in this group; hence, there is no data on these diseases in this article. We did not analyse the patients’ medical records, so we do not have detailed information on where the patients came from (i.e., which clinics). The main reasons for the reported patients to medical entities were: the presence of symptoms of infection, contact with an infected person (including risky sexual contacts) and the willingness to diagnose STI. The analysed data concerned only patients treated for sexually transmitted infections, and people excluded from treatment or treated in several places at the same time were not included. The data obtained from routine surveillance was based on Polish recommendations, which recommend that people who frequently engage in risky sexual behaviour should test for HIV and other sexually transmitted infections every 3 months. They also include women in the first and third trimesters of pregnancy. Due to the data being pseudonymised, the approval of the local Bioethics Committee was not required.

### 2.2. Statistical Analysis

Data were processed using Microsoft Excel 2020 (Microsoft Corporation, Albuquerque, NM, USA) and Statistica 13.0 (StatSoft Company, Hamburg, Germany).

The analysis covered the entire period of 2010–2015. For each disease, the statistics showing the differences in incidence rates between men and women per 100,000 inhabitants were determined by provinces.

The data obtained on the incidence of genital warts and genital herpes were analysed in terms of prevalence among women and men. Information on the gender of HIV patients was not available. The analysis was performed for the entire country, with regional variations by provinces taken into consideration (the analysis was stratified by province). The other stratification was related to gender and age.

The assessment of the statistical significance of differences in the incidence between age groups included data from the entire study period, i.e., 2010–2015, except for groups under 15 years of age, where the small number of cases did not allow the use of statistical inference techniques.

The comparison between regions was carried out using nonparametric methods, in which assumptions about the normality of distribution were not required: the Wilcoxon test and Spearman’s correlation coefficient were used. As for the distribution of the incidence rate for the regions, it was very clearly asymmetric, which excluded the normality of the distribution. The non-normality of the distribution was confirmed by the Shapiro–Wilk test.

Using a logistic regression model, the statistical significance of differences between morbidity in the compared age groups was assessed. Morbidity assessments in each age group were made while controlling for the year of onset, and the values obtained represented the mean annual incidence in 2011–2015.

Odds ratio (OR) and risk ratio (RR) values were generated at a confidence interval (CI) of 95%. Significance was defined by *p* = 0.05 (CI 95%).

## 3. Results

### 3.1. Incidence of Selected Sexually Transmitted Viral Infections in 2010–2015

#### 3.1.1. Genital Herpes

The number of reported cases of genital herpes decreased significantly between 2010 and 2015, i.e., from 791 in 2010 to 339 in 2012 and 619 in 2015.

Nationwide, 3378 patients were diagnosed and treated for genital herpes in venereal clinics during the study period.

The highest incidence of genital herpes in almost all analysed years was found in Mazovia Province and was initially 11.5/100,000 in 2010 and 6.6/100,000 in 2015 (M = 6.1/100,000). The incidence in Poland in the examined period was 1.5/100,000. An increase in incidence was recorded in the south-western provinces of Poland. On the other hand, a decrease in incidence per population was observed in those provinces where the incidence rate was nevertheless higher than the national average, i.e., West Pomeranian, Kuyavian-Pomeranian and Warmia-Masuria provinces. In the provinces located at the eastern border of Poland, the incidence rate of genital herpes in the study period showed a decrease.

The incidence rates of genital herpes were characterised by very pronounced fluctuations from year to year. Aggregated over the years, the highest incidence rate of HSV-2 was observed in Mazovia Province (6.13/100,000), which clearly stood out against other regions. The lowest incidence rate was observed in Greater Poland (0.12/100,000), Pomeranian (0.20/100,000), Świętokrzyskie (0.21/100,000) and Subcarpathian (0.31/100,000) provinces. The analysis showed that the incidence rates were abnormally higher in some provinces compared to other regions. This is indicated by the comparison of mean and median values (Table 1), where in some regions the mean value was significantly higher. As in all analysed years, the median was clearly closer to the minimum value.

#### 3.1.2. Genital Warts

As indicated by the conducted analysis, genital warts are among the most frequently diagnosed and reported sexually transmitted infections in Poland. They are the clinical manifestations of a sexually transmitted infection caused by the human papillomavirus (HPV) types 6 and 11. In the analysed period, 7980 patients of dermatology and venereology outpatient clinics underwent treatment due to genital warts. The highest incidence rates in the country were reported for the Mazovia (46%) and Pomeranian provinces (10%). The lowest number of cases was reported in Subcarpathian and Świętokrzyskie, with 49 and 71 cases, respectively.

The spatial distribution of incidence was similar to that for most STIs, with the highest level in Mazovia Province (11.61/100,000) and the lowest in south-eastern Poland (Subcarpathian Province, 0.38/100,000).

Descriptive statistics for the incidence of genital warts from 2010 to 2015 are presented in Table 1.

#### 3.1.3. HIV

According to general estimates from the National Institute of Public Health, sexual HIV infections occur in 46% of cases (with similar rates for hetero- and homosexual contacts).

In 2015, 1273 newly detected HIV infections were reported under ongoing epidemiological surveillance, with 277 acquired through homosexual sexual contact (22%) and 90 through heterosexual contact (7%). In Poland, there is a persistently high proportion of HIV/AIDS reports in which the probable route of infection was not given (66.9% in 2015).

Nearly half of the AIDS cases in 2015, i.e., 60 cases out of a total of 129, occurred in people who sexually contracted HIV.

The cumulative incidence rate of HIV showed a large spatial variation, i.e., it was even several times higher in the Mazovian Province (5.15/100,000) compared to south-eastern Poland. The prevailing number of infections in the Mazovian Province for HIV was not as clear as it was for the previously analysed STIs.

The values of descriptive statistics of the HIV incidence rate between 2010 and 2015 are presented in Table 1.

### 3.2. Assessment of the Direction of Changes in Incidence between 2010 and 2015

The Spearman rank correlation coefficient was used to assess the systematic changes in incidence rates in individual provinces. The obtained results for individual provinces and diseases are presented in Table 2. Positive correlations mean that the incidence of a given disease tends to increase, and negative when the incidence in subsequent years decreases. The value represents the strength of the correlation.

The analysis showed:

Moderately increasing tendencies for genital warts (R = 0.66) in Lower Silesia;
A statistically significant upward trend in the incidence of genital warts (R = 0.94) and HIV (R = 0.89) in Greater Poland;
A similarly strong upward trend in HIV incidence in the eastern Polish regions, i.e., Podlasie (R = 0.89) and Subcarpathian provinces (R = 0.83).

### 3.3. Incidence by Age Group

Table 3 shows incidence rates for the analysed sexually transmitted viral infections by year and age group.

A compilation of HSV-2 incidence rates by age group in the years analysed showed incidental increases in incidence in other age groups (e.g., in 2012 in infants) as well as years. The peak incidence was at the age of 20–29 years. The incidence rates for genital warts were several times higher in people aged 20–29 years than in other age groups and, at the same time, several times higher than the analysed infections. In the case of HIV infections, the number of cases was several times higher among people aged 20–39 and, to a slightly lesser extent, 40–49 years than in other age groups.

The odds ratio values for contracting the analysed viral sexually transmitted infections in a given age group are presented in Table 4.

A twofold increase in the risk of genital herpes infection was observed in persons over 20 years of age, which gradually decreased in persons over 30 years of age and remained at a level lower than for the general population. The large variation in the incidence of genital warts in relation to age resulted in statistical significance of OR values for each age group. An analogy was noted between the odds ratio values describing the risk of incidence in the age groups. In the case of genital warts, the highest risk was found in the age groups between 20 and 29 years and was associated with up to four times the risk of all STIs. Similarly, the large variation in HIV incidence by age resulted in a statistically significant OR value for each age group relative to the entire population. By far, the highest HIV incidence was seen among those aged 20–29 and 30–39 years. The risk of HIV infection increased tenfold after the age of 20 years compared to people aged 15–19 years. In people under 40 years of age, the risk of infection was almost four times higher than in the general population of the country and twice as high in people under 49 years of age.

### 3.4. Incidence by Gender

Women accounted for less than half of all herpes genitals and genital warts cases in all provinces. The highest proportion of affected women was reported for genital herpes (39.6%) (Table 5).

In the case of genital herpes incidence, regions with significantly higher incidence rates among men compared to women were found. The incidence rates of genital herpes in this group were several times higher among men vs. women in most provinces. In four regions (Lowe Silesia, Lesser Poland, Silesia and Pomeranian Province), men suffered from genital herpes slightly less frequently than women, accounting for 35–45% of patients. In Podlasie and Opole provinces, the percentage of men in the total number of cases was above 80% and was significantly higher than in other regions of Poland. In most regions, a significantly higher incidence of HSV-2 was observed among men (Table 6). The regions where the difference was statistically insignificant included Lublin, Lesser Poland, Pomeranian, Silesian and Świętokrzyskie Province. In Lower Silesia, women were significantly more often affected (RR = 0.65).

The incidence of genital warts was much higher among men in almost all provinces. It ranged from over 75%, i.e., three times more common than in women (West Pomeranian, Lubusz, Świętokrzyskie, Lesser Poland, Subcarpathian and Podlasie provinces), to just over 50% (Lower Silesian or Mazovia provinces), i.e., similar incidence as among women. In all regions of Poland, except for the Lower Silesia Province, there was a statistically significant difference in the incidence of genital warts between men and women. Men were more frequently affected, and the value of the relative risk of incidence in this group was (in some regions) even 3.5 times higher than among women (Table 6).

## 4. Discussion

### 4.1. Genital Herpes

The incidence of genital herpes is not covered by EU surveillance, nor are other viral sexually transmitted diseases, such as genital warts caused by human papillomavirus (HPV6/11), yet they can be a major burden due to their prevalence, duration and health consequences. Based on the developed natural history model for the clinical course of genital ulcers due to HSV-1 and HSV-2 infections, including symptomatic and asymptomatic infections, the population burden of infection was estimated [7]. It is assumed that 417 million people aged 15–49 years (11.3% of the population in this age range) were living with HSV-2 infection worldwide in 2012. Women were almost twice as likely to be infected as men, with incidence rates of 14.8% and 8%, respectively. The number of new infections per year in people of working age was estimated at 19.2 million, representing 0.5% of the population. In Europe, there were 21.7 million women and 9.7 million men with HSV-2. New infections were mainly reported in people aged 15–19 years (0.8%), 20–24 years (0.6%), 25–29 years and 30–34 years (0.5%), with 1% of infections in the population corresponding to an incidence of 1000 cases per 100,000 inhabitants [7].

On the other hand, the data available in Poland included in the analysis show that the number of reported cases of genital herpes is decreasing, which, against the background of global estimates, indicates underreporting. The incidence of herpes in almost the entire country was <1/100,000, except for the Mazovian Province, where it ranged from 4.4 to 11.4/100,000 in the years covered by the study, but with a clear downward trend. Over the entire time period analysed, the number of reported cases decreased by 14% or even 57%, depending on the year, compared to 2010. Nevertheless, the last available global estimate from 2020 [8] showed that the number of HSV infections in 2016 increased to 313 million women and 178 million men (an increase of nearly 18%). In 2016, 3.7 billion people worldwide aged <50 years (i.e., 67%) had oral or genital HSV-1 infection, and 491 million people aged 15–49 years (13%) had genital HSV-2 infection. The incidence of HSV-1 genital ulcers among people of working age (i.e., 15–49 years) was estimated at 122–192 million in 2016, with significant interregional variance. Most genital HSV-1 infections occurred in the Americas [9], Europe [3] and the Western Pacific [7].

The inter-sex differences were mainly due to the difference in the risk of transmission. The prevalence of HSV-2 infection was estimated to be the highest in Africa (44% in women and 25% in men), followed by America (24% and 12%, respectively). The prevalence was also shown to increase with age, which is associated with the persistent nature of infection, and the highest number of newly infected individuals, as in 2012, was reported in the group of adolescent girls [8].

In Poland, the highest incidence occurred in the age groups of 20–29 years and 30–39 years, with an almost identical prevalence, i.e., about 3.3 to 4.4/100,000. In the final year of the study period, an increasing trend of herpes incidence in young people, i.e., under 20 years of age, was noted.

Using a natural history model used for estimating the number of infections and population size, it was estimated that 187 million people (5% of the world population) aged 15–49 years had at least one episode of HSV-related genital ulceration. These were significantly more often caused by the type 2 (4.8% of cases) rather than type 1 virus (0.2%). Genital herpes affected 117 million women (6.4%) and 69 million men (3.6%). In the European region, symptomatic infection occurred in 15 million people, 9 million women and 6 million men, respectively [8]. Interestingly, an analysis of the Polish data showed a higher prevalence among men, i.e., about two times. However, there were border regions (Podlasie and Opole provinces) where the incidence rate among men was five times higher than among women, while in the remaining 14 analysed regions, women were more frequently affected by genital herpes.

The analysed Polish data did not confirm the association of genital herpes incidence with the degree of urbanisation of the region. The frequency of diagnosing genital herpes in Poland was related to the frequency of HPV infections in the regions. However, the conclusion from the analysis may be dictated by the availability of STI diagnostic and therapeutic services in the regions. The presented data on incidence in Poland show the highest incidence of genital herpes in people aged 20–29 years. This trend was maintained throughout the study period; however, a slight increasing trend in HSV incidence was observed in persons aged 30 and 45 years. This increase may be due to the effect of the high prevalence (i.e., percentage of chronically infected persons) proven in the cited studies by other authors.

### 4.2. Genital Warts

Human anal and genital papillomavirus (HPV) is the most common sexually transmitted viral infection worldwide, which can cause malignant or benign skin and mucosal tumours, including anogenital warts (AGW). As in the reviewed literature, genital warts were the most frequently reported STI in Poland. On average, 1300 cases were reported per year, with almost 2000 in 2015. HPV 6 and 11 account for the majority of anogenital warts [10]. They are highly contagious, with approximately 65% of people with an infected partner developing lesions within 3 weeks to 8 months. Although warts are among the most common sexually transmitted infections [11,12], their epidemiology is not well characterised.

A systematic review of 19 studies involving 953,704 men, which was a synthetic analysis of the relationship between the occurrence of male genital cancers with sexual activity and biological mechanisms, proved that 40% of penile carcinomas were found to be associated with HPV infections, particularly HPV 16. Viral infections and orchitis and epididymitis were also among the major sex-related risk factors studied for testicular cancer [13].

In a study to assess the incidence of HPV and other STIs among 177 women with abnormal cervical cytology, it confirmed the high incidence of sexually transmitted diseases in high-risk human papillomavirus (hrHPV)-positive women who are at higher risk of developing cervical disease. At least one hrHPV genotype was in 87% of women; HPV 16 was the most common (25.0%), followed by HPV 31 and HPV 51. Overall positivity for other STDs was 49.2%, with *Ureaplasma parvum* being the most common (39.0%). The obtained results indicate the validity of further research on STI as cofactors with HPV in the carcinogenesis of the cervix [14].

A recent review on the prevalence of genital warts in the world population showed that the prevalence of this disease was less than 5% worldwide and varied depending on the type of data available for statistical analysis. Notably, the prevalence ranged from 0.2% to 5.1% using diagnostic methods, but several times lower prevalence was estimated from data available in administrative or medical databases, i.e., from 0.13% to 0.56% (e.g., medical reports) [15]. Therefore, reports from mandatory reporting and surveillance for the development of health policy and prevention serve primarily as an indicator of trends. They are also useful as an indicator of the need for in-depth population-based studies or studies in critical risk groups.

A meta-analysis of the literature on the prevalence of genital warts from 2001 to 2012 showed an estimated prevalence of AGW (including new and recurrent), regardless of sex, of 160/100,000 in Spain to 289/100,000 in the UK (Me = 194.5). The number of new cases was estimated at 118 in Spain and 205 in North America. The lowest overall incidence was reported for Europe, i.e., 101/100,000. The analysed incidence rate in Poland was unusually lower than the global and other national estimates. Between 2010 and 2015, the maximum incidence ranged only from 5.66 to 20.7/100,000 in Poland. In some, the number of annually diagnosed genital warts cases was only a single incidence. It should be concluded that the epidemiological situation of genital warts in Poland remains unclear. The worldwide incidence of AGW ranged from 103 to 168/100,000 (Me = 137) among men and from 76 to 191/100,000 (Me = 120.5) among women. The incidence of recurrent warts was almost equally high, i.e., 110/100,000 among women and 163/100,000 among men. The incidence peaked in young people under 24 years of age in women and between 25 and 29 years of age in men [16,17,18,19].

Similar estimates, where mandatory surveillance data are not available, have been made in individual countries. Prevalence estimates of anogenital warts in 2011 based on studies from the USA, England and France suggested that genital warts occurred annually in 0.06–0.23% of the population [20]. On the other hand, the incidence of genital warts in the Spanish population aged 14–64 years was estimated at 182/100,000 (203/100,000 in men and 162/100,000 in women), corresponding to 56,446 cases per year. The estimated annual incidence was 118/100,000, including approximately 100/100,000 for women. Extrapolation to the entire Spanish population aged 14–64 years gave a figure of 31,833 men and 24,613 women [18]. The annual burden of HPV infection for the population in Finland ranged from 12,666 to 13,066 new cases of clinical lesions associated with HPV6 or HPV11, implying an incidence of 235/100,000 [21].

Accurate data on the number of cases of genital warts caused by HPV6/11 are available from England. The latest genital warts incidence data indicated a significant 27% decrease between 2009 and 2018, from 77,865 to 57,318 cases. In 2018, over 58,000 screenings were performed in England and 57,318 cases were reported. The incidence was mainly among 20–24-year-olds and 25–34-year-olds, as the number of cases declined markedly in older age groups. Men fell ill slightly more often (58%) than women (42%). Anogenital warts were diagnosed ten times more often in heterosexual men than in men having sexual contacts with men [22]. The genital warts incidence trend established for Poland was different from that in England, as the increase between 2010 and 2015 exceeded 70%. The increase concerned both female and male incidence in parallel, and, as in England, males were more frequently affected in all analysed regions. In Poland, the highest incidence was in people aged 20–29 years, with higher rates for men. In England, 193 cases of genital warts were diagnosed in young girls aged 15–17 years in 2018, and this was a 56% decrease from 2017, a trend repeated among boys of the same age, i.e., a 46% decrease in cases. This was a continuation of the sharp decline observed since 2014, which was largely due to the national HPV vaccination programme [22].

In Germany, on the other hand, the incidence of new and recurrent cases of genital warts was 113.7 and 34.7 per 100,000 women aged 14–65 years, respectively, based on a survey in outpatient specialist practices. The highest incidence was observed in women aged 14–25 years (171/100,000) and for recurrent infections in women aged 26–45 years (53/100,000). The sample size of men in this study was too small to allow a reliable estimate of the incidence of anogenital warts [23]. Another German study that was cross-sectional, with nearly 350,000 men receiving outpatient services between 2013 and 2015 who were screened for STIs, indicated that 1.26% were identified with at least one infection, of which genital warts were the most common (0.64%). The highest incidence was in men aged 21–30 years (i.e., 928, 3.22% of the population) and aged 31–40 years (i.e., 655, 2.04% of the population) [24]. Germany has a relatively low vaccine coverage [25]. Although HPV vaccination for the prevention of cervical cancer was recommended in March 2007 for females aged 12–17 years, in 2008–2009 the vaccine uptake rate was about 40% in females aged 16–18 years and remained at a low 42% vaccination rate in 2017 [26].

The analysis for Poland showed a correlation of the regional prevalence of HPV and HSV infections. Due to the lack of routinely available data on coinfections, it can be assumed that, to some extent, the correlation may have been related to access to medical services and diagnostics of viral infections in the regions (Łódź, Mazovia and Greater Poland provinces).

### 4.3. HIV

In 2015, a total of 153,407 HIV infections were detected in the WHO European Region (17.6/100,000 population). There was a 7% increase in the number of new HIV infections compared to the previous year, with 55,230 officially reported to the joint ECDC/WHO Regional Office for Europe surveillance system. Nearly 100,000 (98,177) infections (i.e., 64%) involved the Russian Federal Scientific and Methodological Centre for the Prevention and Control of AIDS (i.e., 67/100,000) [27]. The epidemiological situation in Russia has a significant impact on the overall situation of the WHO European region. According to the ECDC, 29,747 new HIV infections were detected in 31 EU/EEC countries in 2015 (i.e., 6.3/100,000) [27].

In Poland, from 2015, the number of newly detected infections continued to increase until 2019, i.e., 1615 infections, which was a 27% increase compared to 2015. In 2020, the recorded number of infections decreased, but this data is marked by a bug due to the SARS-CoV-19 pandemic by restricting access to diagnostic tests as a result of the lockdown of health care units [28].

In 2010, more than 27,000 new cases were diagnosed in the EU Member States (5.7/100,000) [29], representing a 10% increase in the number of infections in 2015 compared to 2010. Against the background of Europe, the situation of HIV infections in the period under study was relatively optimistic, with a 5% increase in Poland over the same period, at the same time as a 15% decrease in 2011. However, in recent years, the number of newly detected infections has been increasing significantly. Between 2010 and 2015, HIV detection in Poland was at a much lower level and was about three and one in men and women, respectively [30]. The countries with the highest HIV incidence in 2010 were Estonia (27.8; 372 cases), Latvia (12.2; 274 cases), Belgium (11.0; 1196 cases) and the UK (10.7; 6654 cases). The lowest incidence was reported in Romania (0.7; 152 cases) and Slovakia (0.5; 28 cases), while in Poland it was 4.3/100,000 (i.e., 1207 cases) [29].

In the EU countries in 2010, 11% of HIV infections were diagnosed in people aged 15–24 years [29]. In Poland in 2010, the incidence in 20–29-year-olds was 32.7% and was highest in the 30–39 age group (35.9%). In 2015, the highest incidence was still registered in Estonia (20.6; 270 cases), Latvia (19.8; 393 cases) and Malta (14.2; 61 cases). In this analysis, the incidence rate in Poland was significantly lower (3.3/100,000). Moreover, lower incidence rates were still observed in Slovakia (1.6; 86 cases), as well as in Slovenia (2.3; 48 cases) and the Czech Republic (2.5; 266 cases) [29]. According to the latest available report, Slovenia and Slovakia still have the lowest incidence, while the Czech Republic has seen a sharp increase in the number of newly diagnosed HIV infections, especially among men [29].

For years, Estonia has been the country with the highest HIV incidence in Europe. In 2001, Estonia reached the highest HIV infection rate (105.3 per 100,000 inhabitants) in the European Union, and the reason for this increase was transmission between intravenous drug users. As a result of multiple preventive measures taken by the Estonian government, the number of newly diagnosed cases has been steadily decreasing since 2001. As a result, the incidence of newly diagnosed HIV cases was 24.6 per 100,000 population in 2013 [27,29]. In summary, the age of newly diagnosed was increasing in Estonia over the period 2001–2013, regardless of gender. There are significant gender differences in the transmission route, with a sharp increase in sexual transmission in recent years, especially among women; 30% of people with HIV are simultaneously infected with HCV [27,29]. During the analysed period, no changes in HIV incidence in age groups were noticed in Poland.

In 2008, 6480 newly diagnosed HIV cases were reported in France. After accounting for underreporting, the actual number of infections was estimated at 6940 (6200–7690, CI: 95%) and the incidence at 17/100,000 persons per year, including: 3550 (3040–4050 cases) new infections in heterosexuals (9/100,000), 3320 (2830–3810) in MSM (1006/100,000) and 70 (0–190) among injecting drug users (86/100,000) [30]. Based on HIV surveillance information, i.e., CD4 cell levels in people with newly diagnosed HIV infection in Italy in 2016, the actual number of people living with HIV was estimated. According to these estimates, the undiagnosed HIV population was 13,729–16,475. The proportion of undiagnosed infections was higher among people under 25 years of age (25–28%), MSM (16–19%) and foreign-born people (16–19%), while the proportion was low among injecting drug users (3%) [31].

### 4.4. Limitations of the Study

The study has certain limitations. First of all, the analysed data concerned the number of people treated in outpatient specialist medical centres in the field of venereology on the basis of a medical diagnosis. There were in public statistics no reporting guidelines verifying the basis of diagnosis, such as symptoms or laboratory tests. Secondly, we did not analyse the patients’ medical records. The localisation of illness lesions of viral sexually transmitted infections was not included in the study, because the data collection system of information for STIs in Poland does not take into account such types of data. Thirdly, data on STVIs come from two independent reporting sources in Poland; hence, the data format was heterogeneous in terms of age groups, and information on gender was not available due to the possibility of anonymous reporting of newly detected HIV infections.

## 5. Conclusions

The incidence of genital warts increased statistically significantly during the study period, while the incidence of genital herpes decreased. The incidence of HIV infections remained stable. The highest incidence of all analysed sexually transmitted viral infections in Poland was found in the age group of 20–29 years. Higher incidence rates among people aged 40–49 years were reported for HIV and genital warts. The incidence of genital herpes infections increased in the group of 15–19-year-olds. Men were significantly more likely to be diagnosed with genital warts. Genital herpes occurred regardless of gender.

The worsening epidemic situation with respect to sexually transmitted infections, the inefficiency of the current surveillance system and the reduction in funding for diagnosis and prevention, combined with inadequate legal solutions, make it necessary to undertake new legal and organisational measures aimed at improving the reproductive health in Poland in terms of sexually transmitted infections.

## Figures and Tables

**Table 1:** Distribution of descriptive statistics of incidence per 100,000 inhabitants for the analysed sexually transmitted infections between 2010 and 2015.

	Year	M	Me	SD	Min.	Max.
Genitals herpes (*p*_2015 vs. 2010_ = 0.5695)	2010	1.38	0.34	2.88	0.00	11.48
	2011	1.30	0.51	2.10	0.00	8.14
	2012	0.90	0.68	0.73	0.00	2.63
	2013	1.04	0.43	1.36	0.07	4.95
	2014	1.02	0.50	1.28	0.13	4.39
	2015	1.11	0.59	1.63	0.04	6.64
Genital warts (*p*_2015 vs. 2010_ = 0.0386 *)	2010	2.55	2.13	2.51	0.33	10.24
	2011	2.19	2.04	1.46	0.38	5.66
	2012	2.40	2.20	1.73	0.61	7.47
	2013	3.37	2.77	2.79	0.23	11.49
	2014	3.14	1.90	3.39	0.47	14.04
	2015	3.77	2.77	4.71	0.28	20.73
HIV (*p*_2015 vs. 2010_ = 0.0061 *)	2010	1.76	1.58	1.07	0.43	4.73
	2011	2.75	2.35	1.54	0.85	6.89
	2012	2.27	2.27	1.13	0.55	5.32
	2013	2.52	2.07	1.26	1.03	5.89
	2014	2.32	2.13	0.91	1.11	4.16
	2015	2.71	2.84	1.37	0.42	5.27

Abbreviations: M—mean, Max.—maximum, Me—median, Min.—minimum, SD—standard deviation, *p*_2015 vs. 2010_—assessment of the significance of differences in the incidence rate between 2010 and 2015, * statistically significant result (*p* ≤ 0.05).

**Table 2:** Spearman rank correlation coefficients of STI for provinces.

Province	Sexually Transmitted Infections		
	Genital Herpes	Genital Warts	HIV
Lower Silesia	0.20	0.66	0.37
Kuyavian-Pomeranian	0.03	−0.20	0.03
Lublin Province	−0.49	0.09	−0.09
Lubusz Province	0.54	0.37	0.43
Lodz Province	0.89 *	0.94 *	0.43
Lesser Poland	0.26	−0.66	0.54
Mazovia Province	−0.49	0.83 *	−0.54
Opole Province	0.49	0.60	0.20
Subcarpathian Province	−0.49	−0.20	0.83 *
Podlasie Province	0.94 *	0.60	0.89 *
Pomeranian	−0.37	0.43	0.20
Silesia	0.77	−0.20	0.49
Świętokrzyskie Province	−0.09	0.14	0.31
Warmia-Masuria Province	−0.31	0.66	−0.43
Greater Poland	0.58	0.94 *	0.89 *
West Pomeranian Province	−0.94 *	−0.66	0.60

* statistically significant correlations (*p* ≤ 0.05).

**Table 3:** Incidence of selected STVIs in age groups by year per 100,000 population.

Sexually Transmitted Infection	Age Group (Years)	Incidence Rate per 100,000 Population					
		2011	2012	2013	2014	2015	*p*_2015 vs. 2010_
Genital herpes	<1	0.00	4.75	0.28	0.00	0.00	1.0000
	1–9	0.00	0.11	0.03	0.11	0.08	0.0873
	10–14	1.87	0.16	0.05	0.00	0.17	<0.001 *
	15–19	3.15	0.36	1.84	0.34	2.48	0.1903
	20–24	4.50	2.11	4.14	3.29	3.36	0.0390 *
	25–29	4.48	2.41	3.72	3.52	4.59	0.8349
	30–44	1.85	1.16	1.14	1.46	1.86	0.9694
	45–64	1.03	0.53	0.81	0.84	1.10	0.6563
	≥65	0.71	0.35	0.65	0.78	1.17	0.0133 *
Genital warts	<1	0.00	0.26	1.67	0.27	0.55	0.1431
	1–9	0.09	0.11	0.17	0.31	0.39	0.0089 *
	10–14	0.10	0.21	0.05	0.22	0.17	0.5993
	15–19	1.73	1.26	2.45	2.31	3.29	0.0011 *
	20–24	8.07	8.53	11.84	9.99	14.89	<0.001 *
	25–29	9.04	9.25	15.32	12.50	18.54	<0.001 *
	30–44	3.35	4.07	5.38	5.78	7.70	<0.001 *
	45–64	0.95	0.98	1.00	1.51	2.12	<0.001 *
	≥65	0.39	0.58	0.49	1.06	1.25	<0.001 *
HIV	<1	0.77	0.79	0.83	0.00	1.38	0.4203
	1–14	0.02	0.07	0.06	0.04	0.13	0.0331 *
	15–19	0.52	0.72	0.56	0.74	1.01	0.0623
	20–29	7.38	5.76	7.93	6.51	8.41	0.0522
	30–39	7.69	6.22	7.81	6.60	7.23	0.3545
	40–49	4.14	3.60	4.06	3.81	4.26	0.7537
	50–59	1.24	0.99	1.30	1.33	1.24	0.9899
	≥60	0.47	0.25	0.41	0.23	0.33	0.1410

*p*_2015 vs. 2010_—assessment of the significance of differences in the incidence rate in 2010 and 2015, * statistically significant result (*p* ≤ 0.05).

**Table 4:** Odds ratio of incidence of selected sexually transmitted viral infections by age group.

Sexually Transmitted Infection	Age Group (Years)	Disease Risk Assessment		
		OR	OR (95% CI)	*p*
Genital herpes	15–19	1.00	0.88–1.13	0.9693
	20–24	2.11	1.94–2.30	<0.001 *
	25–29	2.26	2.08–2.45	<0.001 *
	30–44	0.90	0.84–0.98	0.0093 *
	45–64	0.52	0.48–0.57	<0.001 *
	≥65	0.45	0.40–0.50	<0.001 *
Genital warts	15–19	0.64	0.58–0.72	<0.001 *
	20–24	3.13	2.96–3.31	<0.001 *
	25–29	3.78	3.60–3.98	<0.001 *
	30–44	1.54	1.46–1.61	<0.001 *
	45–64	0.38	0.36–0.41	<0.001 *
	≥65	0.22	0.20–0.25	<0.001 *
HIV	15–19	0.34	0.28–0.40	<0.001 *
	20–29	3.60	3.40–3.82	<0.001 *
	30–39	3.66	3.45–3.88	<0.001 *
	40–49	2.02	1.88–2.16	<0.001 *
	50–59	0.64	0.58–0.70	<0.001 *
	≥60	0.17	0.15–0.20	<0.001 *

* statistically significant result (*p* ≤ 0.05).

**Table 5:** Summary incidence for diseases included in the analysis in the female group.

Disease	% of Women in the Total Number of Cases in 2010–2015				
	M	Me	SD	Min.	Max.
Genitals herpes	39.6%	37.7%	13.9%	17.9%	62.3%
Genital warts	31.6%	32.1%	7.6%	22.6%	49.6%

**Table 6:** Comparison of incidence of selected sexually transmitted viral infections by sex between 2010 and 2015.

Sexually Transmitted Disease	Province	Women		Men		M/F RR (95% CI)	*p*
		N	I	N	I		
Genital herpes	Lower Silesia	71	4.70	43	3.07	0.65 (0.45–0.95)	0.0265 *
	Kuyavian-Pomeranian	71	6.58	130	12.81	1.95 (1.46–2.60)	<0.001 *
	Lublin Province	45	4.04	57	5.44	1.35 (0.91–1.99)	0.1351
	Lubusz Province	32	6.11	52	10.45	1.71 (1.10–2.66)	0.0155 *
	Lodz Province	41	3.11	69	5.75	1.85 (1.25–2.72)	0.0016 *
	Lesser Poland	54	3.13	35	2.15	0.69 (0.45–1.05)	0.0822
	Mazovia Province	881	31.82	1066	41.96	1.32 (1.20–1.44)	<0.001 *
	Opole Province	5	0.96	23	4.72	4.91 (1.87–12.93)	0.0004 *
	Subcarpathian Province	10	0.92	30	2.88	3.13 (1.53–6.40)	0.001 *
	Podlasie Province	7	1.14	31	5.31	4.65 (2.05–10.55)	0.0001 *
	Pomeranian	15	1.28	12	1.07	0.84 (0.39–1.80)	0.6531
	Silesia	114	4.78	85	3.82	0.80 (0.60–1.06)	0.1149
	Świętokrzyskie Province	6	0.92	10	1.61	1.75 (0.63–4.80)	0.2746
	Warmia-Masuria Province	50	6.77	82	11.56	1.71 (1.20–2.43)	0.0026 *
	Greater Poland	8	0.45	18	1.07	2.38 (1.03–5.46)	0.0357 *
	West Pomeranian Province	70	7.94	155	18.52	2.33 (1.76–3.09)	<0.001 *
Genital warts	Lower Silesia	122	8.07	124	8.85	1.10 (0.85–1.41)	0.4700
	Kuyavian-Pomeranian	131	12.14	269	26.51	2.18 (1.77–2.69)	<0.001 *
	Lublin Province	108	9.70	163	15.57	1.60 (1.26–2.04)	0.0001 *
	Lubusz Province	60	11.45	185	37.19	3.25 (2.43–4.34)	<0.001 *
	Lodz Province	82	6.22	165	13.75	2.21 (1.69–2.88)	<0.001 *
	Lesser Poland	102	5.90	298	18.30	3.10 (2.47–3.88)	<0.001 *
	Mazovia Province	1594	57.57	2107	82.94	1.44 (1.35–1.54)	<0.001 *
	Opole Province ^(#)^	40	7.69	86	17.66	2.30 (1.58–3.34)	<0.001 *
	Subcarpathian Province	12	1.10	37	3.55	3.22 (1.68–6.17)	0.0002 *
	Podlasie Province	71	11.58	215	36.84	3.18 (2.43–4.15)	<0.001 *
	Pomeranian	72	6.13	150	13.42	2.19 (1.65–2.90)	<0.001 *
	Silesia	222	9.32	539	24.25	2.60 (2.22–3.04)	<0.001 *
	Świętokrzyskie Province	17	2.61	54	8.70	3.33 (1.93–5.74)	<0.001 *
	Warmia-Masuria Province	127	17.19	253	35.68	2.07 (1.68–2.57)	<0.001 *
	Greater Poland	87	4.89	165	9.79	2.00 (1.54–2.60)	<0.001 *
	West Pomeranian Province	73	8.28	250	29.87	3.60 (2.77–4.67)	<0.001 *

N—number of cases in 2010–2015, I—incidence per 100,000 inhabitants; RR—relative risk of male versus female incidence; *p*—assessment of statistical significance of differences in incidence between men and women; ^(#)^ RR estimated by means of a specific adjustment on a numerical value equal to 0; * statistically significant result (*p* ≤ 0.05).

## Data Availability

Data are available upon reasonable request.
